# Comprehensive transcriptome analysis of mouse embryonic stem cell adipogenesis unravels new processes of adipocyte development

**DOI:** 10.1186/gb-2010-11-8-r80

**Published:** 2010-08-03

**Authors:** Nathalie Billon, Raivo Kolde, Jüri Reimand, Miguel C Monteiro, Meelis Kull, Hedi Peterson, Konstantin Tretyakov, Priit Adler, Brigitte Wdziekonski, Jaak Vilo, Christian Dani

**Affiliations:** 1 Université de Nice Sophia-Antipolis, Institut Biologie du Développement et Cancer, CNRS UMR 6543, Faculté de Médecine Pasteur, 28 avenue de Valombrose, 06108 Nice Cedex 2, France; 2 Institute of Computer Science, University of Tartu, Liivi 2, 50409, Tartu, Estonia; 3 Quretec, Ülikooli 6a, 51003 Tartu, Estonia; 4 Institute of Molecular and Cell Biology, University of Tartu, Riia 23b, 51010 Tartu, Estonia

## Abstract

**Background:**

The current epidemic of obesity has caused a surge of interest in the study of adipose tissue formation. While major progress has been made in defining the molecular networks that control adipocyte terminal differentiation, the early steps of adipocyte development and the embryonic origin of this lineage remain largely unknown.

**Results:**

Here we performed genome-wide analysis of gene expression during adipogenesis of mouse embryonic stem cells (ESCs). We then pursued comprehensive bioinformatic analyses, including *de novo *functional annotation and curation of the generated data within the context of biological pathways, to uncover novel biological functions associated with the early steps of adipocyte development. By combining in-depth gene regulation studies and *in silico *analysis of transcription factor binding site enrichment, we also provide insights into the transcriptional networks that might govern these early steps.

**Conclusions:**

This study supports several biological findings: firstly, adipocyte development in mouse ESCs is coupled to blood vessel morphogenesis and neural development, just as it is during mouse development. Secondly, the early steps of adipocyte formation involve major changes in signaling and transcriptional networks. A large proportion of the transcription factors that we uncovered in mouse ESCs are also expressed in the mouse embryonic mesenchyme and in adipose tissues, demonstrating the power of our approach to probe for genes associated with early developmental processes on a genome-wide scale. Finally, we reveal a plethora of novel candidate genes for adipocyte development and present a unique resource that can be further explored in functional assays.

## Background

Obesity has become a major public health problem for industrialized countries. This pathology is associated with an increased risk of metabolic troubles, such as type 2 diabetes, cardiovascular diseases, and certain types of cancers. Obesity is the result of an imbalance between energy intake and expenditure and is often characterized by an increase in both adipocyte size (hypertrophia) and number (hyperplasia). Besides the clinical importance of obesity, we still have limited information regarding the origin and the development of fat tissues.

Adipogenesis is generally described as a two-step process. The first step consists of the generation of committed adipocyte precursors (or preadipocytes) from mesenchymal stem cells (MSCs), while the second step involves the terminal differentiation of these preadipocytes into mature, functional adipocytes. By definition, MSCs are endowed with self-renewal properties and differentiation potentials towards all mesenchymal cell types, while preadipocytes have lost the ability to differentiate into mesenchymal derivatives other than adipocytes. The differentiation of preadipocytes into adipocytes has been extensively studied *in vitro *using preadipocyte cell lines that were selected from disaggregated mouse embryos or adult adipose tissue for their ability to accumulate cytoplasmic triacylglycerols [1-3]. These cell lines are believed to be faithful models of preadipocyte differentiation and they have provided important insights into the transcriptional control of the late steps of adipogenesis. In contrast, the early steps of adipocyte development remain largely unknown. Although there have been attempts to characterize the distinct cellular intermediates between MSCs and mature adipocytes, such studies have been hampered by the lack of specific cell surface markers to identify and prospectively isolate these cells *in vivo*. The recent identification and isolation of subpopulations of white adipocyte progenitors in the vasculature of mouse adipose tissues, however, opens new avenues for the understanding of fat cell formation and their modulation in pathological contexts [4,5].

Until now, knowledge about mesenchymal cell fate decisions has been mostly derived from studies on the immortalized mouse stromal cell line C3H10T1/2, or mesenchymal precursor populations isolated from adult tissues. However, these cellular systems are not informative for the developmental origin of MSCs and adipocytes. Instead, the embryo might constitute a more suitable source of cells to address this issue and elucidate the exact pathways and intermediates between the embryonic stem cell and the mature adipocyte. In particular, mouse embryonic stem cells (mESCs) have provided an invaluable tool to model the earliest steps of adipocyte development *in vitro*. mESCs are proliferating, pluripotent stem cells that can be propagated indefinitely *in vitro *in the presence of leukemia inhibitory factor (LIF) [6,7]. When transplanted into a mouse blastocyst, mESCs integrate into the embryo and contribute to all cell lineages, including germ cells [8]. Similarly, when mESCs are cultured without leukemia inhibitory factor on a non-adherent surface, they aggregate to form embryoid bodies (EBs) containing ectodermal, mesodermal, and endodermal derivatives, thus offering a unique cell culture model to study the earliest steps of mammalian development [9]. Directed differentiation of mESCs towards the adipocyte lineage was first accomplished by Dani *et al. *[10], who showed that functional adipocytes could be obtained by exposing EBs to an early and transient treatment with retinoic acid (RA). To dissect out the molecular mechanisms involved in the early steps of adipogenesis, we have recently performed a small-scale drug screening in mESCs using synthetic retinoids as well as pharmacological inhibitors of several signaling pathways [11]. We have demonstrated that retinoic acid receptor β (RARβ) activation is both necessary and sufficient for the commitment of mESCs to the adipocyte lineage. Conversely, pharmacological inhibition of the glycogen synthase kinase 3 (GSK3) completely inhibits RARβ-induced adipogenesis in mESCs, uncovering the requirement of active GSK3 in this process. The induction of mESC differentiation upon single or combined treatment with RARβ agonist and GSK3 inhibitors therefore provides a selective set of screening conditions to uncover the genes involved in the early steps of adipogenesis.

Here, we have used this powerful comparative system to perform a large-scale gene expression profiling of mESC adipogenesis, using a high throughput Affymetrix platform. We then pursued in-depth comprehensive bioinformatics analyses, including *de novo *functional annotation and curation of the generated data within the context of biological pathways, to unravel several important biological functions associated with the early steps of adipocyte development in mESCs. Finally, we provide a basis for a more comprehensive understanding of how transcriptional regulatory networks might govern these early steps by combining detailed gene regulation studies with *in silico *analysis of transcription factor (TF) binding sites (TFBSs).

## Results and discussion

### Large-scale gene expression profiling of mESC adipogenesis

**Figure 1:**
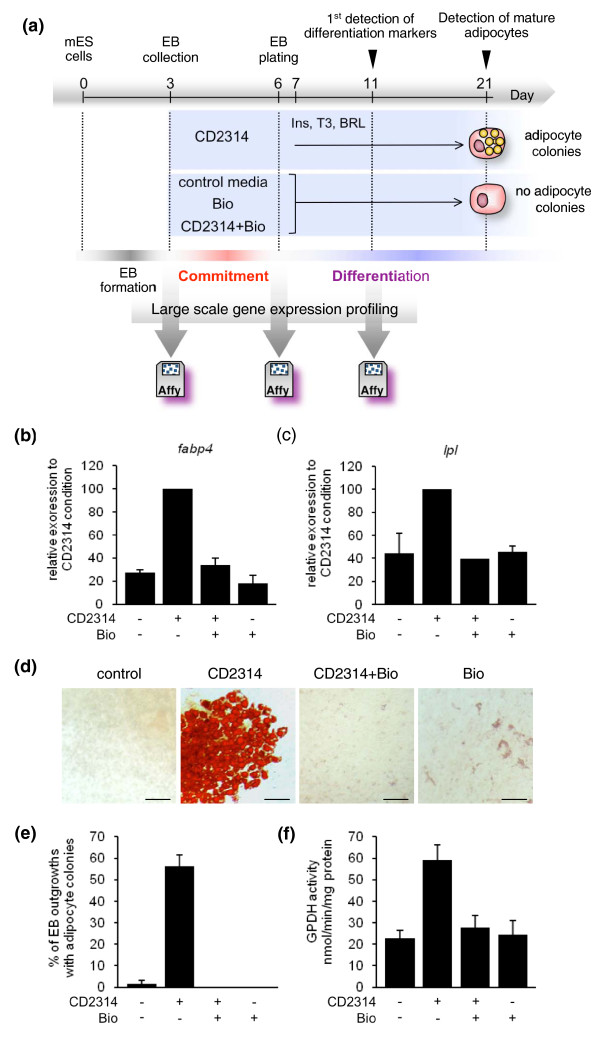
**Experimental strategy used for large-scale gene expression profiling of mESC adipogenesis**. **(a) **Summary scheme of our experimental design. Adipocyte commitment was selectively stimulated through exposure of EBs to CD2314, or repressed through the addition of the GSK3 inhibitor BIO, or both compounds, between day 3 and day 6. Adipocyte terminal differentiation was further induced by addition of the adipogenic compounds insulin (Ins), triiodothyronine (T3), and rosiglitazone (BRL) from day 7 to day 21. For microarray analysis, samples were generated before (day 3), right after (day 6), or 5 days after (day 11) exposure to control medium, CD2314, BIO, or CD2314+BIO and analyzed using Mouse Genome 430 2.0 Affymetrix Arrays. Expression of adipocyte differentiation markers could first be detected at day 11, while mature adipocytes were detected at day 21. **(b, c) **Quantification of *Fabp4 *or *Lpl *RNA expression by quantitative PCR at day 11. The relative expression level of each RNA upon CD2314 stimulation was considered as 100%. **(d) **Oil red O staining of mature adipocyte colonies at day 21. Scale bar: 50 μM. **(e) **Quantification of the percentage of EB outgrowths with adipocyte colonies at day 21. **(f) **Quantification of glycerol-phosphate dehydrogenase (GPDH) activity at day 21. Here and in the following figures, data are displayed as mean values ± standard error of the mean of at least three independent experiments.

To uncover the genes involved in the early steps of adipogenesis, we compared gene expression profiles of mESCs in which adipocyte development was selectively stimulated through early exposure to the RARβ agonist CD2314, or repressed through the addition of the GSK3 inhibitor BIO, or both compounds. A summary scheme of this strategy is given as Figure 1a. As previously described, stimulation of mESCs with CD2314, from day 3 to 6 after EB formation, was sufficient to induce adipocyte development in this system, as monitored by the expression of adipocyte differentiation-specific markers such as *fatty acid binding protein 4 *(*Fabp4*) and *lipoprotein lipase *(*Lpl*) genes (Figure 1b, c), oil red O staining of triglycerides in mature adipocytes (Figure 1d, e), and glycerol-phosphate dehydrogenase (GPDH) activity (Figure 1f). Conversely, adipogenesis was strongly inhibited in untreated, as well as Bio- and CD2314+Bio-treated cultures (Figure 1). We therefore generated gene expression profiles of mESCs before (day 3) or immediately after (day 6) exposure to these signals, assuming that potential regulators and markers of the early steps of mesenchymal/adipocyte commitment would be enriched in the stimulatory condition. To uncover factors potentially involved in later stages of adipocyte differentiation, we also monitored gene expression at day 11, which represents the earliest time of appearance of adipocyte differentiation-associated factors, such as *Fabp4*, in the mESC culture system (Figure 1e). We identified gene expression profiles using Affymetrix GeneChip Mouse Genome 430 2.0 microarrays. The raw data can be obtained from ArrayExpress (accession number [E-TABM-668]).

To define genes that are selectively associated with the early steps of adipocyte development in mESCs, we compared the expression levels of CD2314-treated mESCs with those of untreated, Bio- and CD2314+Bio-treated cells at days 6 and 11. We selected only the genes that were either significantly up- or downregulated in CD2314-treated mESCs compared to all three other non-adipogenic conditions. Of the 16,810 genes and expressed sequence tags that are represented on the chips, 500 fulfill these criteria, corresponding to 342 EnsEMBL unique genes. These transcripts were then organized into five clusters that reflect the time when they are differentially expressed during mESC differentiation (Additional file 1). Clusters 1 and 2 contain the genes that are upregulated or downregulated by CD2314 at day 6, respectively; clusters 3 and 4 contain the genes that are upregulated or downregulated by CD2314 at day 11, respectively; and cluster 5 contains genes that are modulated by CD2314 at both day 6 and day 11, and thus encompasses potential candidate genes for both early and later steps of adipocyte development.

We next validated our microarray data by examining the expression levels of 30 representative genes by quantitative real-time PCR (qPCR) in three independent experiments (Additional file 2). These genes encompassed several biological categories, such as cell surface and extracellular matrix components, TFs, and signal transduction and metabolism-associated molecules. As indicated in Additional file 2, the expression profiles of 29 of these genes gave comparable patterns to the microarray analysis, i.e. a validation rate of 96%.

### Adipocyte development is associated with several important biological functions in mESCs

**Figure 2:**
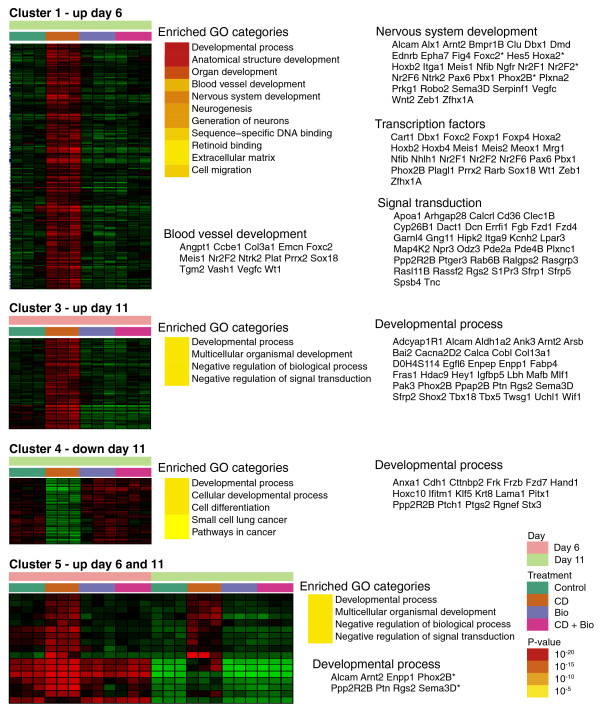
**Functional annotation and Gene Ontology category enrichment in mESC adipogenesis-associated genes**. Functional annotation and enrichment of GO categories in clusters 1 to 5. Heatmap diagrams with time points and treatments are represented on the left. Hypergeometric GO enrichment *P*-values reported by g:Profiler are represented using a yellow-to-brown color scale. All the statistically significant results are shown, with the exception of cluster 1, where we picked only the most relevant GO categories out of all significant results. For some GO categories, we also point out the corresponding genes within the cluster. Genes related to neural crest development are indicated with an asterisk. Note that no significant enrichments were detected for cluster 2 (down-regulated genes, day 6).

**Figure 3:**
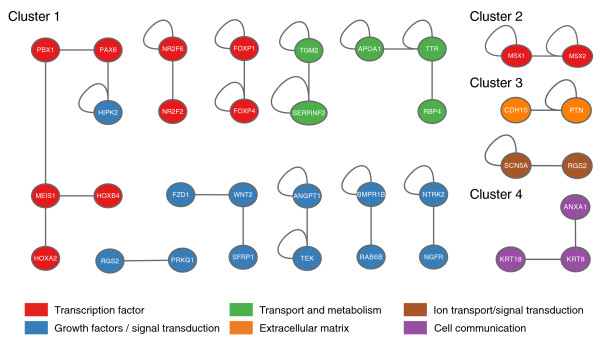
**Protein-protein interaction in mESC adipogenesis-associated clusters**. Modules of interacting proteins found in clusters 1 to 5, as detected by GraphWeb software. Colored circles represent proteins and gray lines denote physical PPIs, while circular loops denote interactions within the same species of molecules (for example, homodimers). Nodes are colored according to the functional role of corresponding proteins.

Functional annotation of individual clusters was performed using g:Profiler, a web interface that captures Gene Ontology (GO), pathways, TFBSs and microRNA sequence enrichment down to the individual gene level (Figure 2) [12]. We also predicted protein-protein interactions (PPIs) in each cluster using the manually curated collection of PPIs from the Human Protein Reference Database [13] and applying a conservative strategy (Figure 3; see Materials and methods for more details). Extensive inspection of these clusters highlighted several important biological functions associated with adipocyte development in mESCs, which we detail below.

#### Early steps of adipocyte development are coupled to blood vessel formation in mESCs

The first phase of adipogenesis, which begins right after CD2314 exposure (day 6 of differentiation, cluster 1), is characterized by a dramatic enrichment in genes involved in developmental processes, such as organ and anatomical structure development (Figure 2). Among them, many are known to regulate blood vessel morphogenesis, such as *angiopoietin 1 *(*Angpt1*), its receptor *endothelial-specific receptor tyrosine kinase *(*Tek*), *vascular endothelial growth factor C *(*Vegfc*), *disintegrin-like and metallopeptidase with thrombospondin type 1 motif 1 *(*Adamts1*) and the TF *forkhead box C2 *(*Foxc2*). A close spatial and temporal relationship between adipocyte and blood vessel formation exists during fetal development. Blood vessels and first formed adipocytes appear coincidentally and are always found in close association *in vivo*, so that a common precursor for adipocytes and endothelial cells has been suggested, although never formally isolated [14-16]. The prevalence of vasculature-associated genes in CD2314-regulated cluster 1 suggests that, similarly to normal development, the early steps of adipocyte formation in mESCs are coupled to blood vessel morphogenesis. Interestingly, an elegant genetic lineage-mapping study recently shed new light on the interplay between the adipocyte and the endothelial lineages by showing that white adipocyte progenitors reside in the mural compartment of the adipose vasculature in mice [4]. The observation that the mESC culture system might allow the development of both adipose cells and their potential vascular niche opens exciting perspectives for the prospective isolation and the biochemical characterization of these newly identified adipocyte progenitors by offering an abundant source for these cells.

#### Early steps of adipocyte development are coupled to neural development in mESCs

Another striking observation that arises from the functional annotations of both clusters 1 and 5 (Figure 2) is the enrichment in neural development-associated genes at both day 6 and day 11 of mESC adipogenesis. This group includes genes like the neurotrophin receptors *neurotrophic tyrosine kinase*, *receptor*, *type 2 *(*Ntrk2*) and *nerve growth factor receptor *(*ngfr*) [17], *roundabout homolog *(*robo2*) [18], the TFs *hairy and enhancer of split 5 *(*Hes5*) [19] and *paired box gene 6 *(*Pax6*) [20], which are all known to play important functions in the control of mammalian neurogenesis and axon guidance. Interestingly, this group also contains genes involved in the development of the neural crest, such as *homeobox A2 *(*Hoxa2*) [21], *paired-like homeobox 2b *(*Phox2B*) [22], *semaphorin 3 D *(*Sema3D*) [23] and *endothelin receptor type B *(*Ednrb*) [24] (indicated by an asterisk in Figure 2). The neural crest comprises a transient cell population of vertebrate embryos that generates the peripheral nervous system, pigment cells, most of the craniofacial skeleton, as well as other derivatives [25]. These data indicate that the early steps of adipocyte formation might be closely associated with nervous system/neural crest development. In accordance with these findings, we have demonstrated that, during normal mouse development, a subset of adipocytes in the cranial region of the body is generated by the neural crest, rather than by mesodermal progenitors, classically thought to be at the origin of this lineage [26]. Furthermore, we have shown that adipocytes obtained from mESCs upon RA treatment are mostly derived from the neuroectoderm and that this phenomenon is associated with a precocious upregulation of neural crest markers [26]. The neural origin of adipocytes generated by embryonic stem cells exposed to RA has been confirmed by Takashima *et al. *[27], who also used an elegant approach to demonstrate that the earliest wave of MSC production in the mouse embryo is generated from the neuroepithelium, and not the mesoderm. The results presented here corroborate these findings and indicate that genomics data can be successively mined to unravel plausible biological functions. They further indicate that RARβ might mediate RA effects on neural and adipocyte development in mESCs.

#### Early steps of adipocyte development involve major changes in cell signaling components: analysis of the Wnt pathway

**Figure 4:**
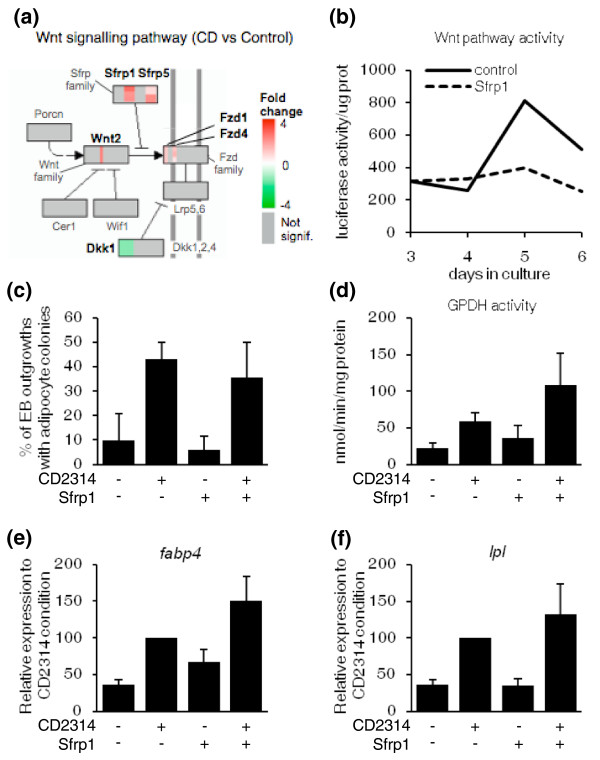
**Status of the Wnt pathway and effect of exogenous addition of sFRP-1 during mESC adipogenesis**. **(a) **Stationary view of the KEGG canonical Wnt signaling pathway at day 6 of mESC differentiation in cells treated with CD2314 compared to untreated cells. Genes in the pathway are represented as colored rectangles, each stripe within a rectangle representing one gene member of the same family. Fold changes in RNA levels in the CD2314 condition compared to untreated control determine the colors on a red-to-green scale, with red meaning induction, green meaning repression, and grey meaning no significant variation. **(b) **Wnt pathway activity in differentiating mESCs stably transfected with the TOP-FLASH reporter construct. EBs were left untreated (control, solid line) or incubated with 100 ng/ml of recombinant sFRP-1 (secreted frizzled-related protein-1; dashed line) from days 3 to 6. **(c-f) **Effect of exogenous addition of sFRP-1 on adipocyte development. EBs were incubated with CD2314 and sFRP-1, alone or in combination, from days 3 to 6, and adipocyte development was assessed as in Figure 1.

The early steps of adipocyte development in mESCs are illustrated by an enrichment in a wide variety of extracellular factors and signal transduction components (Figure 2), suggesting that differentiating cells become endowed with an array of receptors and accessory molecules to fine-tune the activation of the major signal transduction pathways. This event, in conjunction with the induction of tissue-specific TFs (see next section), might allow immature stem or precursor cells to launch lineage-specific differentiation programs. Of note, both clusters 1 and 3, which correspond to genes upregulated during adipocyte development, contain several members of the Wnt pathway (Figure 2; Additional file 1), which has previously been identified as a major regulator of preadipocyte differentiation *in vitro *and *in vivo *(for a review, see [28]). To examine the action of this individual pathway, we used KEGGanim, a recently developed web-based tool that allows the visualization of dynamic changes in genetic, signaling or metabolic pathways in time-related or treatment-related animations [29]. Genes in the pathway are represented as colored rectangles and expression values or fold changes determine the colors on a red-to-green scale, allowing intuitive visual analysis of the selected pathway. Figure 4a depicts a stationary view of the Kyoto Encyclopedia of Genes and Genomes (KEGG) canonical Wnt signaling pathway at day 6 in cells treated with CD2314, compared to untreated cells. To avoid confusion, only the genes significantly affected by these treatments have been colored and annotated on the pathway. These include *wingless-related MMTV integration site 2 *(*Wnt2*), its receptors *frizzled homolog *(*Fzd*) *1 *and *4*, as well as *dickkopf homolog 1 *(*Dkk1*) and *protein phosphatase 2 regulatory subunit B delta isoform *(*Ppp2r2d*). Of note, both *secreted frizzled-related proteins *(*sFRP*) *1 *and *5*, two extracellular inhibitors of the Wnt pathway, are strongly upregulated by CD2314 at day 6, suggesting that an inhibition of the Wnt pathway activity, besides its demonstrated role in adipocyte terminal differentiation, might also be involved in the early steps of adipogenesis (Figure 4a). Interestingly, forced expression of sFRP-1 in 3T3-L1 cells stimulates preadipocyte differentiation, and sFRP-1-deficient male mice have diminished body fat [30,31]. In addition, elevated expression of sFRP-5 has been associated with fat mass expansion in diet-induced obese mice [32].

To clarify the role of sFRPs in the early steps of adipocyte development, we assessed the effect of exogenous addition of sFRP-1 on differentiating mESCs. To measure Wnt pathway activity, we used mESCs stably transfected with the TOP-FLASH reporter construct, which contains the Firefly luciferase reporter gene under the control of TCF/LEF (T-cell-specific transcription factor/lymphoid enhancer binding factor) response elements (TCF/LEF being the main transcriptional effectors of the Wnt pathway). As expected, sFRP-1 addition between days 3 and 6 of EB formation inhibited Wnt pathway activity (Figure 4b). However, sFRP-1 addition, alone or in combination with CD2314, had no significant effect on adipocyte formation, GPDH activity, or the expression of adipocyte-differentiation markers (Figure 4c-e). Therefore, inhibiting the Wnt pathway activity through addition of exogenous sFRP-1 was not sufficient to drive adipocyte development in mESCs. In accordance with other recent observations, these results suggest that although RARβ and active GSK3 are required for adipocyte formation in mESCs, they are likely to be acting through a Wnt pathway-independent mechanism [11].

### *In silico *gene regulation analysis provides a basis for the understanding of transcriptional control of adipocyte development

The differentiation of preadipocytes into adipocytes is regulated by an extensive network of TFs that coordinate the expression of several genes essential for the acquisition of mature fat-cell characteristics. Among them, Peroxisome proliferator-activated receptor γ (PPARγ) and CCAAT-enhancer-binding proteins (C/EBPs) are considered as master regulators of the entire terminal differentiation process (for a review, see [33]). In contrast, the transcriptional processes controlling the conversion of mesenchymal precursors to preadipocytes are largely unknown. To provide a basis for a more comprehensive understanding of how transcriptional control governs these early steps in mESCs, we used a combination of computational and experimental approaches.

#### Analysis of the expression of TFs associated with mESC adipogenesis during mouse embryogenesis and in mouse adipose tissues

**Figure 5:**
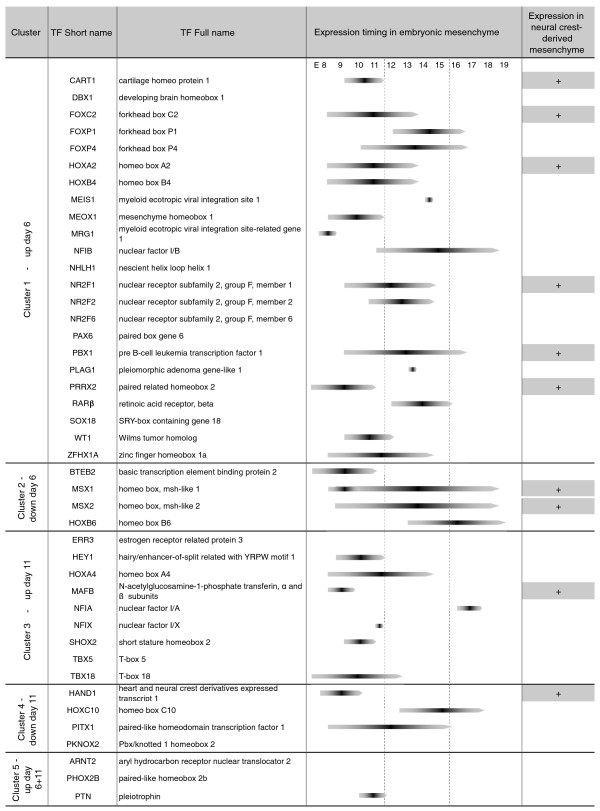
**Analysis of the expression of mESC adipogenesis-associated TFs in embryonic mesenchyme**. CD2314-modulated TF-encoding genes were extracted from clusters 1 to 5 and their expression was checked in embryonic mesenchyme using the Mouse Genome Informatics web tool. When available, indications about their timing of expression in embryonic mesenchyme, and their prevalence in neural-crest-derived mesenchyme, are also shown. E, embryonic day.

We first used information from the TRANSFAC database [34] to screen clusters 1 to 5 for the presence of TF-encoding genes (Figures 2 and 5). Interestingly, the early steps of adipocyte development in mESCs are characterized by a dramatic gain of scores of TFs, many of which have been associated with embryonic development and patterning, such as genes of the homeo box (HOX) and forkhead box (FOX) families. A good proportion of these TFs also belong to the Nuclear receptor (Nr) gene family (Figure 5, cluster 1). Several of these TFs have been shown to act as critical regulators of adipogenesis, such as FOXC2 [35,36], NR2F1 [37,38] and NR2F2 [39,40]. Others, such as genes of the HOX network, have been found to be expressed in human white and brown adipose tissues, as well as in the 3T3-L1 preadipocyte cell line [41,42]. Conversely, the group of TFs significantly downregulated by CD2314 (Figure 5, cluster 2) include homeobox, msh-like 2 (MSX2), which has been shown to inhibit adipogenesis in the C3H10T1/2 mesenchymal cell line by binding to C/EBPα and inhibiting its ability to transactivate the *Pparγ *promoter [43,44].

In addition to TFs known to participate in adipogenesis and adipose function, we also identified several TFs with no previous link to adipocyte biology. To assess the relevance of the TFs differentially expressed in mESCs for mesenchymal and adipocyte formation during normal development, we used the Mouse Genome Informatics (MGI) web tool, together with extensive literature curation, to analyze the reported expression of such TFs during mouse embryogenesis. In particular, we had a closer look at mesenchymal tissues, since the adipocyte lineage originates from mesenchymal precursors. As reported in Figure 5, a large proportion (77%) of the TFs present in clusters 1 to 5 were detected in mesenchymal compartments between day 7 and day 18.5 of mouse embryonic development. Interestingly, some of them (23%) were specifically associated with neural crest-derived mesenchyme, again suggesting that adipocytes developing from mESCs do so, at least in part, through a neural crest pathway.

**Figure 6:**
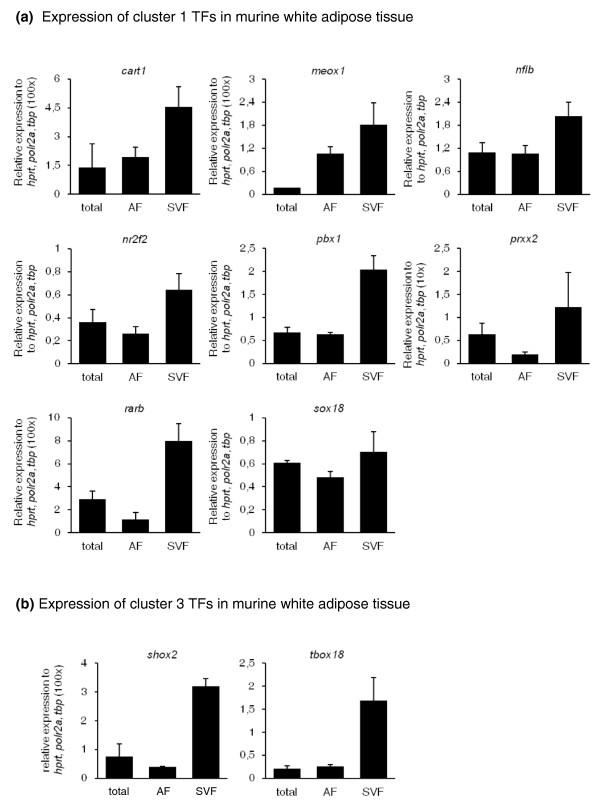
**Expression of mESC adipogenesis-associated TFs in murine white adipose tissue**. **(a, b) **TFs whose expression was upregulated by CD2314 during mESC adipogenesis were selected from cluster 1 (a) and from cluster 3 (b) and their expression was then checked by qPCR in total or fractionated periepidymal WAT isolated from 10-week-old mice. For simplicity, for those genes whose relative expression was weaker than the reference genes, relative expression values were multiplied by 10 or 100 as indicated on the y-axis.

To gain further insight into the relevance of some of these TFs during mouse adipogenesis, we next assessed for their expression in fractionated white adipose tissue (WAT) from young mice. We reasoned that good candidates for the regulation of the early steps of adipogenesis *in vivo *would likely be expressed in the stromal vascular fraction (SVF), which contains, among other cell types, adipocyte progenitors, rather than in the adipocyte fraction (AF), which encompasses only mature adipocytes. Out of eleven TFs studied, ten can be detected in mouse WAT (Figure 6). Seven of these ten TFs were enriched in the SVF fraction, three TFs were expressed similarly in both SVF and AF, while no TF was expressed only in the AF fraction. All together, these results indicated that the vast majority of the TFs associated with the early steps of adipocyte development in mESCs are also expressed in mesenchymal areas during mouse embryogenesis and/or in the adipose progenitor compartment of mouse adipose tissues. The curated data that we present here should therefore provide a comprehensive resource for studies into the transcriptional control of early adipocyte development, which can be further explored in functional assays.

#### *In silico *analysis of TFBS enrichment in CD2314-modulated clusters

**Figure 7:**
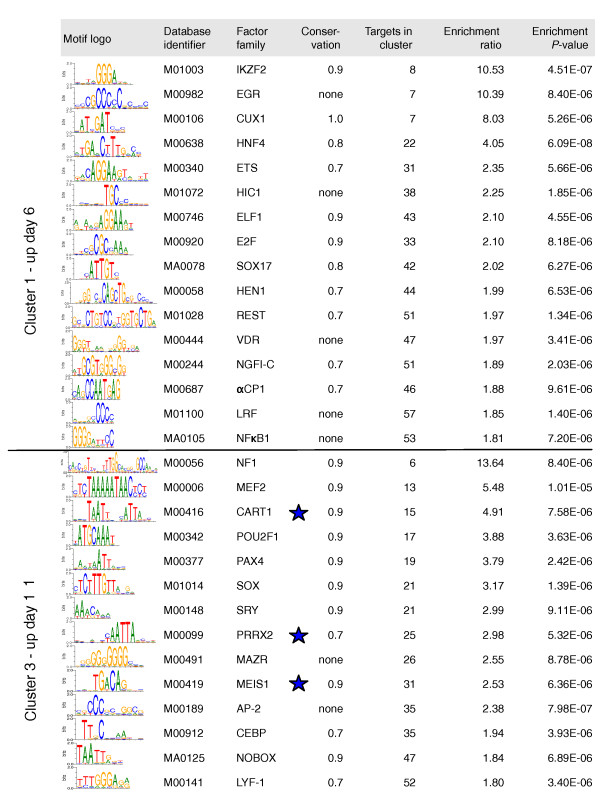
**Statistically over-represented TFBSs in CD2315-regulated gene promoters**. *In silico *analysis was performed to predict TFBS enrichment in clusters 1 to 5. The motifs with identifiers starting with M0 and MA0 are from TRANSFAC and JASPAR databases, respectively. For each motif, information is given about the TF family known to bind this motif, the conservation level that gave the best over-representation, the number of gene promoters displaying the motif in the cluster, the number of times the motif was enriched from the comparison with all other murine gene promoters, and the *P*-value before Bonferroni correction. TFs that were also differentially expressed during mESC adipogenesis are marked with stars.

Genes co-expressed at the early steps of mesenchymal and adipocyte development in mESCs may be co-regulated by the same TFs. It follows that TFBSs responsible for driving these coordinated gene expression programs are likely to be overrepresented in the *cis*-acting regions of those genes. To investigate this hypothesis, we used a computational approach to identify DNA motifs that are statistically overrepresented in the putative promoter and enhancer regions of genes specifically modulated by CD2314 (see Materials and methods). Using this approach, we detected 16 significantly enriched motifs in cluster 1, and 14 in cluster 3, both encompassing CD2314-upregulated genes (Figure 7).

Some of the enriched motifs highlighted by our analysis bind to TFs already known to exert a pro-adipogenic effect in various cellular models of adipocyte differentiation. For instance, Leukemia/lymphoma-related factor (LRF) has been show to be expressed in human and mouse adipocyte precursors, where it might promote differentiation by blocking cell cycle progression [45]. Similarly, Early growth response protein-2 (EGR2, or KROX20), is induced early during 3T3-L1 adipogenesis and promotes C/EBPβ expression, while decreasing its expression reduces the ability of these cells to differentiate [46]. Finally, C/EBP family members act as master regulators of adipocyte differentiation both *in vitro *and *in vivo *[33]. Together, these data suggest that TFBS enrichment analysis may constitute a very useful approach to unravel new transcriptional networks involved in the early steps of mESC adipogenesis.

Besides binding sites for TFs known to participate in adipogenesis, we also identified several motifs that suggested novel factors in adipocyte biology. For instance, the genes associated with adipocyte development in mESCs were enriched for a motif bound by three members of the Activator protein 2 (AP-2) family of TFs [47]. In mice, these TF genes (*AP-2α*, *AP-2β *and *AP-2γ*) are co-expressed in neural crest cells, the peripheral nervous system, as well as facial and limb mesenchyme, where they play crucial roles during development [18,48]. Mutation of *AP-2α *predominantly affects the cranial neural crest and the limb mesenchyme, leading to profound disturbances of facial and limb development. Together, these data place the AP-2 family members as interesting candidates for the regulation of adipocyte generation through the neural crest developmental pathway, which has been shown to account for the generation of cephalic WAT in mouse [26].

Finally, we performed an integrated study compiling TFBS enrichment results, gene expression profiling and PPI analysis. Interestingly, three motifs enriched in cluster 3 (day 11) correspond to binding sites for TFs upregulated at day 6 of mESC adipogenesis (indicated by a star in Figure 7): Cartilage homeo protein 1 (CART-1), Paired related homeobox 2 (PRRX2), and myeloid ecotropic viral integration site 1 (MEIS1) (Figure 5). As revealed by our PPI analysis (Figure 3), MEIS1 physically interacts with HOXB4, HOXA2 and Pre B-cell leukemia transcription factor 1 (PBX1) in a large transcriptional network also involving PAX6 and homeodomain interacting protein kinase 2 (HIPK2), and all these regulators exhibit transcriptional upregulation at day 6 of adipogenesis. To rule out the possibility that this predictive candidate regulatory network might represent a 'noisy artifact' from our transcriptomic study, we examined the expression of its components during mESC adipogenesis at the protein level. As shown in Additional file 3, the interacting partners of this regulatory network could all be detected by western blot in the mESC system. Interestingly, most of them showed the predicted, specific upregulation at day 6 of adipocyte development. In addition, MEIS1, PBX1 and HOXB4 were also upregulated at day 11 of adipocyte development, when enrichment in TFBSs for MEIS1 could be predicted from our *in silico *analysis, reinforcing the idea that the integrated approach presented here could be very useful to unravel important novel regulators of adipocyte development. Several biochemical and genetic approaches have shown that MEIS1, PBX1 and HOX factors associate in trimeric, DNA-binding transcriptional complexes to modulate gene expression during early embryonic development and organogenesis [49,50]. Additional studies should now explore the precise role of this network in regulating the early steps of adipocyte commitment.

## Conclusions

In the present study, we have used a unique cell model and genome-wide analysis of gene expression to uncover the signaling and transcriptional networks underlying the early steps of adipocyte development, a process that remains largely unknown. Although expression profiling using microarrays has been reported for different adipocyte cell models, no such study aiming at uncovering the early steps of adipocyte formation in mESCs had been conducted so far [51]. Our comprehensive and unbiased approach resulted in the identification of 500 transcripts differentially expressed during mESC adipogenesis, a large proportion of which had no previous link to adipocyte biology. Therefore, these results considerably increase existing knowledge of adipocyte development. Besides providing an unprecedented look at transcriptional changes over the course of adipocyte development in mESCs, our study supports several important biological findings.

First, adipocyte development in mESCs is coupled to blood vessel morphogenesis and neural development, closely mimicking normal mouse development. The observation that the mESC culture system might not only allow the generation of adipose stem cells, but also of their potential vascular niche, opens new avenues for the study of adipose stem cell biology and their close relationship with the endothelial lineage.

Second, the early steps of adipocyte formation involve important changes in cell signaling, including components of the Wnt pathway. Of these, we have further investigated the role of a single candidate, sFRP-1. Functional validation studies provided evidence that this factor is not sufficient to drive adipocyte commitment in the mESC system, suggesting that more complex regulatory mechanisms are needed to allow immature stem cells to launch adipocyte lineage-specific differentiation programs.

Third, adipocyte development in mESCs is regulated by an extensive network of TFs, which might coordinate the expression of genes essential for the acquisition of mesenchymal and adipocyte characteristics. By combining computational and experimental approaches, we show here that a large proportion of these TFs are also expressed in mesenchymal areas during mouse embryogenesis and in mouse adipose tissues, therefore demonstrating the power of our system to probe for genes associated with early developmental processes on a genome-wide scale. In addition, using *in silico *analysis of the promoters of co-expressed genes, we uncover further putative transcriptional networks that could drive these coordinated gene expression programs.

Finally, we reveal a plethora of novel candidate genes for adipocyte development, and present a unique and comprehensive resource that can be further explored in functional assays.

## Materials and methods

### mESC culture and induction of adipocyte development

CGR8 mESCs [52] were used in this study and were propagated as previously described [10,53]. For EB formation, mESCs were cultivated in aggregates as previously described [10,53]. From days 3 to 6, EBs were incubated in control medium alone or in the presence of either the RARβ-selective agonist CD2314, the inhibitor of the glycogen synthase kinase (2'Z,3'E)-6-bromoindirubin-3'-oxime (BIO) or both compounds, as previously described [11]. At day 6, EBs were allowed to settle onto gelatin-coated plates. From day 7 onward, EB outgrowths were cultured in the presence of 85 nM bovine insulin, 2 nM triiodothyronine, and 0.5 μM rosiglitazone (BRL4953), a PPARγ agonist. Media were changed every 2 days until day 21. CD2314 was kindly provided by Professor Pierre Chambon (IGBMC; Illkirch, France) and rosiglitazone was a gift of GlaxoSmithKline, Marly le Roy, France. The remaining compounds were bought from Sigma-Aldrich, Lyon, France. For sFRP experiments, differentiating EBs were treated with 100 ng/ml of recombinant sFRP-1 (R&D Systems, Lille, France) daily from days 3 to 6.

### Assessment of adipocyte differentiation

Lipid droplets were visualized after Oil Red O staining as described previously [10,53]. Enzymatic activity of GPDH, an adipocyte-specific enzyme, was measured as previously described [10]. Expression of adipocyte differentiation markers such as *Fabp4 *and *Lpl *was measured using qPCR as described below.

### White adipose tissue isolation and fractionation

Periepidymal WAT was obtained from 10-week-old C57Bl/6J male mice in accordance with the French and European regulations for the care and use of research animals. Adipose tissues were digested using collagenase (300 U/ml in phosphate-buffered saline, 2% bovine serum albumin, pH 7.4) for 45 minutes under constant shaking. Following removal of the floating mature AF, the lower layer containing the SVF was successively filtrated through 100, 70, and 40 μm sieves, centrifuged (200g, 10 minutes) and resuspended in erythrocyte lysis buffer (Sigma) for 1 minutes. SVF cells were then resuspended in phosphate-buffered saline/2% fetal calf serum and processed for RNA extraction together with the AF.

### Microarray experimental design, RNA isolation and microarray hybridization

Samples were generated from mESCs before (day 3) or immediately after (day 6) exposure to control medium, CD2314, BIO, or CD2314+BIO. Samples were also taken at day 11 after exposure to these four conditions. A summary scheme of this strategy is given in Additional file 1. The total number of experimental conditions was then nine, each performed in three separate biological repeats. Total RNA from embryonic stem cells or their derivatives was isolated using the RNeasy Mini Kit from Qiagen (Courtaboeuf, France) and treated with RNase-free DNase I (5 U/100 μg of nucleic acids, Sigma). Biotinylated cRNA was prepared according to the standard Affymetrix protocol. In brief, double-stranded cDNA was synthesized from 10 μg total RNA using the SuperScript Choice System from Invitrogen (Cergy Pontoise, France) and the Affymetrix T7-(dT)_24 _primer, which contains a T7 RNA polymerase promoter attached to a poly-dT sequence. Following a phenol/chloroform extraction and ethanol precipitation, the cDNA was transcribed into biotin-labeled cRNA using the Retic Lysate IVT™ kit (Ambion Inc., Woodward Austin, TX, USA). cRNA products were purified using the RNeasy kit (Qiagen) and fragmented to an average size of 30 to 50 bases according to Affymetrix recommendations. Fragmented cRNA (15 μg) was used to hybridize the Mouse Genome 430 2.0 Array for 16 hours at 45°C. The arrays were washed and stained in the Affymetrix Fluidics Station 400 and scanned using the Hewlett-Packard GeneArray Scanner G2500A. Image data were analyzed with the GeneChip^® ^Operating Software (GCOS) using Affymetrix default analysis settings. After quality control tests, arrays were normalized by the log scale robust multi-array analysis [54]. The raw data can be obtained from ArrayExpress (accession number [E-TABM-668]).

### Statistical analyses

To identify genes that display a CD2314-specific expression profile, we performed *t*-tests with each other condition on the same time point. The final clusters are composed of genes that were either up- or downregulated significantly in all comparisons. We used false discovery rate (*P *= 0.05) as the significance threshold in each of the tests. The statistical analysis was performed using the Limma package from Bioconductor [55].

All biological quantification data are shown as mean values ± standard error of the mean of at least three independent experiments and tested statistically using two-tailed Student's *t*-test or Z-test for location, unless otherwise indicated.

### Functional annotations, protein-protein interactions, and pathway animations

For functional annotations of selected groups of genes, we used the recently developed g:Profiler web toolkit [12]. To assess PPIs in selected clusters, we downloaded the manually curated collection of human PPIs from the Human Protein Reference Database [13]. Corresponding mouse proteins were retrieved with the g:Orth tool [12]. We used a conservative strategy to map orthologs and only accounted for interactions where both interacting proteins in human had exactly one corresponding protein in mouse. The PPIs in our clusters were extracted with the GraphWeb tool [56] by setting the input to the Human Protein Reference Database interactions and the Network Neighbourhood to the list of differentially expressed genes of a selected cluster (distance = 0). To better visualize dynamic changes in entire genetic and signaling cellular circuits, we used the KEGGanim web toolkit [29], which involves a set of animations based on pathways from KEGG [57].

### *In silico *prediction analysis of transcription factor binding sites

To predict TFs potentially involved in mESC adipogenesis, we performed *in silico *analysis of TFBSs in the promoter sequences of CD2314-modulated genes (clusters 1 to 5). Because sequence-specific TFs show a marked increase in binding across the region that encompasses each transcription start site [58], we focused our analysis on the 2,000 bp upstream and 1,000 bp downstream of each transcription start site. We used the UCSC genome database (mm8 release) to extract these sequences, with transcription start site defined by track RefSeq genes [59,60]. In case several promoters had more than 1,500 bp overlapping, we randomly discarded all but one of the promoters. In the obtained promoters, we searched for TFBS enrichment, for example, TFBSs that were occurring more often than expected from the comparison with all other mouse gene promoters. For the source of TFBSs, we used the position weight matrices from the TRANSFAC (version 11.4) [34] and JASPAR [61] databases. As the first step of the analysis, each position weight matrix was scored in every promoter by taking the highest scoring hit within the promoter using the Storm software [62]. While scanning the promoter for the best match, we only considered the regions that are conserved according to the UCSC Euarchontoglires conservation track. For conservation, we used four different thresholds (0.7, 0.8, 0.9 and 1.0) plus we also performed the analysis with no conservation required. In the latter case, each promoter was scored by averaging the three highest scoring hits in the promoter, instead of a single hit. For both clusters 1 and 3, a position weight matrix was considered interesting if the proportion of promoters scoring over some threshold was significantly higher in the promoters of this list than among all other mouse promoters. The threshold was optimized for the fold-change in the proportion, while requiring the Bonferroni-corrected hypergeometric *P*-value to be below 0.01. We discarded the results with fold-change less than 1.8 times.

### Quantitative real-time-PCR

Total RNA was extracted using TRI-Reagent™ kit (Euromedex, Souffelweyersheim, France) according to the manufacturer's instructions and RT-PCR analysis was conducted as described previously. All primers sequences are detailed in Additional file 4. For qPCR, the final reaction volume was 25 μl, including specific primers (0.4 μM), 10 ng of reverse transcribed RNA and 12.5 μl SYBR green master mix (Eurogentec, Angers, France) qPCR conditions were as follows: 2 minutes at 50°C, 10 minutes at 95°C, followed by 40 cycles of 15 s at 95°C, 1 minute at 60°C. Real-time PCR assays were run on an ABI Prism 7700 real-time PCR machine (PerkinElmer Life Sciences, Courtaboeuf, France). Relative gene expression was calculated by the dCT method and normalized to the geometric mean of the expression of three reference genes (*βactin*, *gapdh *and *tbp*).

## Abbreviations

AF: adipocyte fraction; AP: Activator protein; BIO: (2'Z,3'E)-6-bromoindirubin-3'-oxime; C/EBP: CCAAT-enhancer-binding protein; EB: embryoid body; FABP4: fatty acid binding protein 4; FOXC2: forkhead box C2; GO: Gene Ontology; GPDH: glycerol-phosphate dehydrogenase; GSK: glycogen synthase kinase; HIPK: homeodomain interacting protein kinase; HOXA2: homeobox A2; KEGG: Kyoto Encyclopedia of Genes and Genomes; LPL: lipoprotein lipase; MEIS1: myeloid ecotropic viral integration site 1; mESC: mouse embryonic stem cell; MSC: mesenchymal stem cell; PAX6: paired box gene 6; PBX1: Pre B-cell leukemia transcription factor 1; PPARγ: Peroxisome proliferator-activated receptor γ; PPI: protein-protein interaction; qPCR: quantitative real-time PCR; RA: retinoic acid; RAR: retinoic acid receptor; sFRP: secreted frizzled-related protein; SVF: stromal vascular fraction; TF: transcription factor; TFBS: transcription factor binding site; WAT: white adipose tissue; Wnt: wingless-related MMTV integration site.

## Authors' contributions

NB conceived the study, organized its design and its coordination, collected data, and wrote the manuscript. RK performed statistical analyses, and participated in functional annotation, conception of the bioinformatics studies, and design of the figures. JR conceived the g:Profiler web toolkit, carried out PPI analysis, participated in the conception and the design of the bioinformatics studies, and reviewed the manuscript. MCM carried out all the gene expression analysis and contributed to mESC culture and sFRP experiments. RK, JR and MCM contributed equally to this work. MK and HP conceived and designed the *in silico *prediction analysis of TFBSs. KT participated in the design of the *in silico *gene regulation analysis. PA conceived the KEGGanim web toolkit. BW provided technical assistance for mESC culture and RNA isolation. JV and CD participated in the design of the study, in data collection, and provided financial support. All authors read and approved the final manuscript.

## Supplementary Material

Additional file 1 **Hierarchical clustering of mESC adipogenesis-associated-genes into five expression clusters**. Genes that were expressed at significantly different levels in CD2314-treated embryonic stem cells compared to untreated, BIO- and CD2314+Bio-treated cells at day 6, day 11, or both time points were selected and organized into five expression clusters, depicted as Heatmapper pictures here. Clusters 1 and 3 contain the genes that are upregulated by CD2314 at day 6 and day 11, respectively. Clusters 2 and 4 contain the genes that are downregulated by CD2314 at day 6 and day 11, respectively. Cluster 5 contains the genes that are upregulated by CD2314 at both day 6 and day 11. Click here for file

Additional file 2 **Expression validation of microarray candidate genes by qPCR in mESCs**. Representative genes encompassing several biological categories were selected from clusters 1 and 3 (CD2314-upregulated genes at day 6 and day 11, respectively) and their expression was assessed during mESC adipogenesis by qPCR. Click here for file

Additional file 3 **Protein expression validation of MEIS1-associated transcriptional complex in mESCs**. Cell extracts were generated at various time points before (day 0 and 3) or after (day 6 and 11) induction of adipocyte development by CD2314. The protein levels of HIPK2, PAX6, MEIS1 HOXB4, HOXA2, PBX1, and HSP60 were then assessed by conventional western blot analysis. Rabbit polyclonal antibodies were from Abcam (Paris, France; HIPK2, HOXB4, HOXA2) or Millipore (Molsheim, France; PAX6). Goat polyclonal antibody against HSP60 was from Santa Cruz Biotechnology, Inc. (Heidelberg, Germany) and was used to control for protein loading. Mouse antibodies against PBX1 and MEIS1 were generous gifts from M Cleary (Stanford University). Click here for file

Additional file 4 **Primer pairs used for qPCR**. Click here for file
