# Supplementary material for: Comprehensive transcriptome analysis of mouse embryonic stem cell adipogenesis unravels new processes of adipocyte development
Source: Genome Biol. 2010 Aug 3;11(8):R80. doi: 10.1186/gb-2010-11-8-r80 (PMC2945782; doi:10.1186/gb-2010-11-8-r80)
Supplement: Additional file 2 — Expression validation of microarray candidate genes by qPCR in mESCs. Representative genes encompassing several biological categories were selected from clusters 1 and 3 (CD2314-upregulated genes at day 6 and day 11, respectively) and their expression was assessed during mESC adipogenesis by qPCR. [file gb-2010-11-8-r80-S2.PPT]

## Slide 1
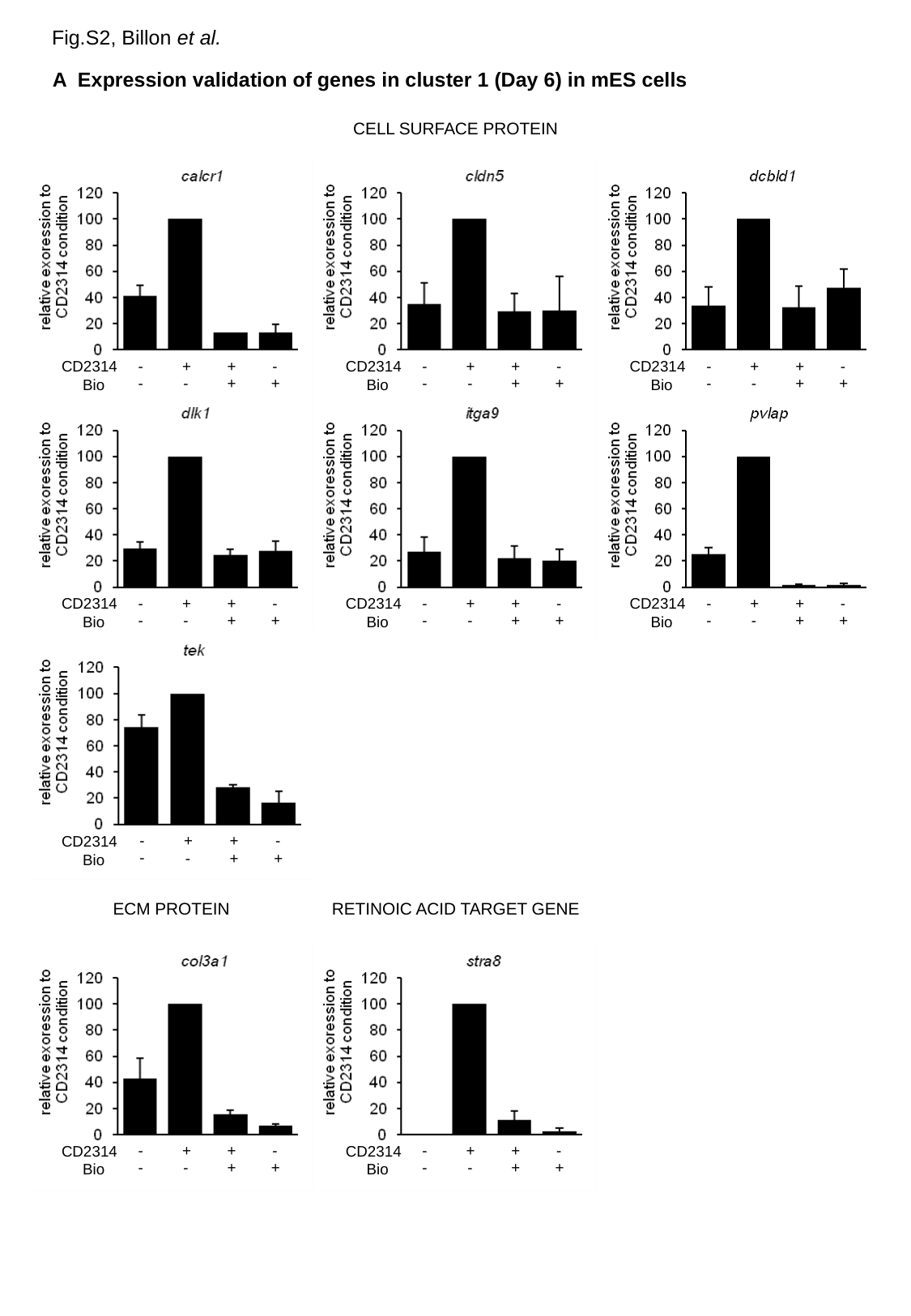

Fig.S2, Billon et al.
A Expression validation of genes in cluster 1 (Day 6) in mES cells
CELL SURFACE PROTEIN
CD2314
Bio
-
+
+
-
CD2314
Bio
-
+
+
-
CD2314
Bio
-
+
+
-
-
-
+
+
-
-
+
+
-
-
+
+
CD2314
Bio
-
+
+
-
CD2314
Bio
-
+
+
-
CD2314
Bio
-
+
+
-
-
-
+
+
-
-
+
+
-
-
+
+
-
+
+
-
CD2314
Bio
-
-
+
+
ECM PROTEIN
RETINOIC ACID TARGET GENE
CD2314
Bio
-
+
+
-
CD2314
Bio
-
+
+
-
-
-
+
+
-
-
+
+

## Slide 2
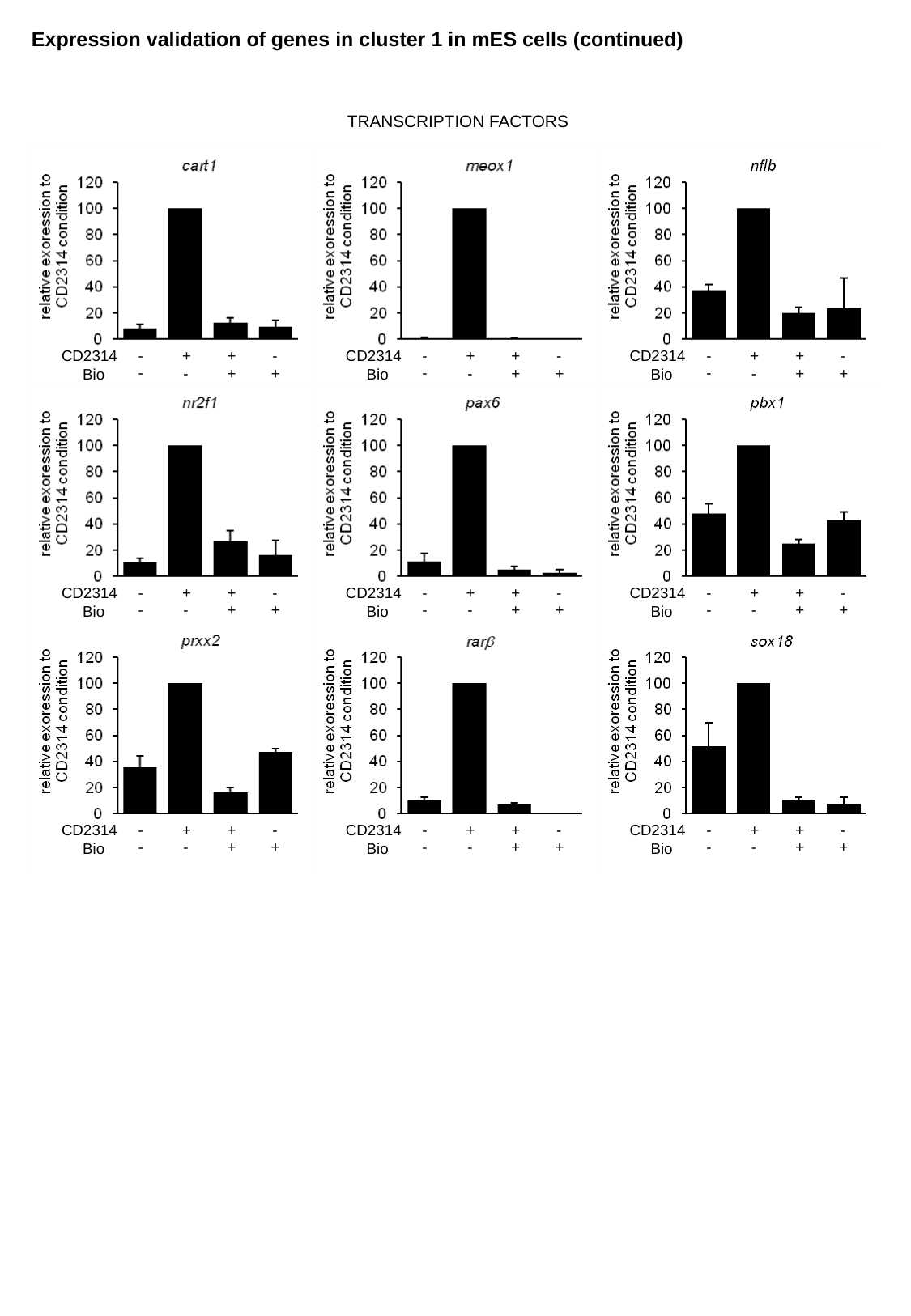

Expression validation of genes in cluster 1 in mES cells (continued)
TRANSCRIPTION FACTORS
CD2314
Bio
-
+
+
-
CD2314
Bio
-
+
+
-
CD2314
Bio
-
+
+
-
-
-
-
-
+
+
-
+
+
-
+
+
CD2314
Bio
-
+
+
-
CD2314
Bio
-
+
+
-
CD2314
Bio
-
+
+
-
-
-
+
+
-
-
+
+
-
-
+
+
CD2314
Bio
-
+
+
-
CD2314
Bio
-
+
+
-
CD2314
Bio
-
+
+
-
-
-
+
+
-
-
+
+
-
-
+
+

## Slide 3
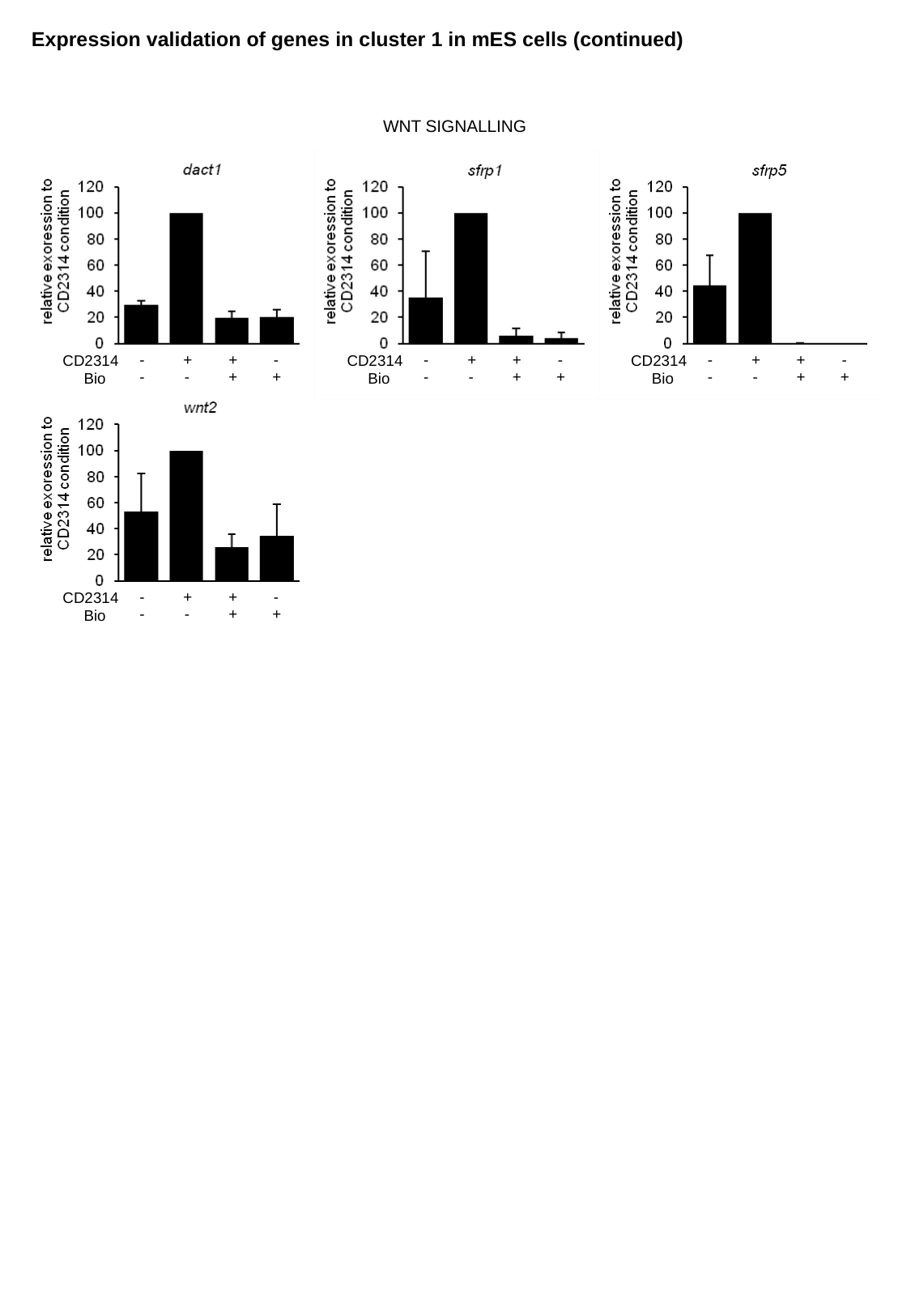

Expression validation of genes in cluster 1 in mES cells (continued)
WNT SIGNALLING
CD2314
Bio
-
+
+
-
CD2314
Bio
-
+
+
-
CD2314
Bio
-
+
+
-
-
-
+
+
-
-
+
+
-
-
+
+
CD2314
Bio
-
+
+
-
-
-
+
+

## Slide 4
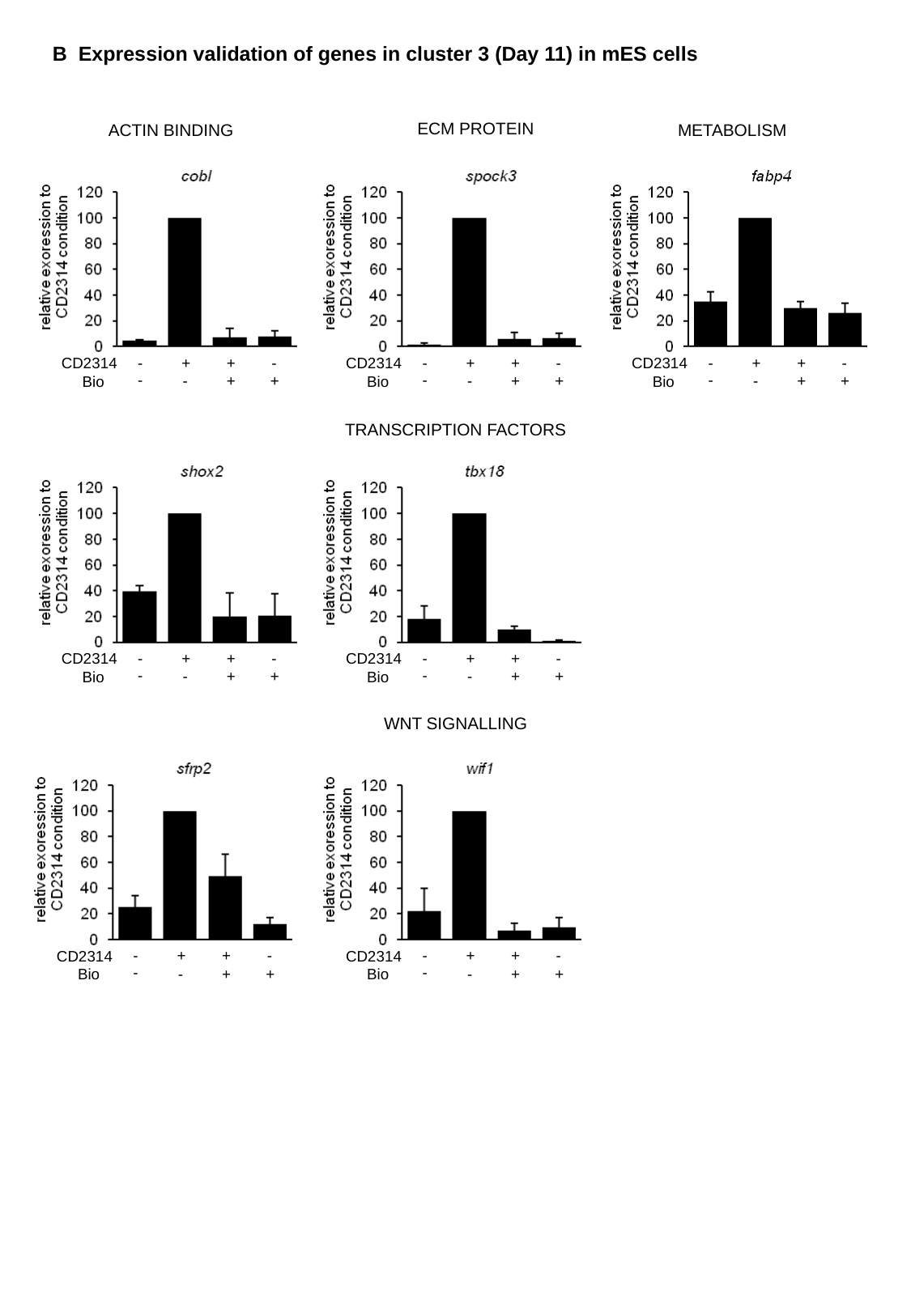

B Expression validation of genes in cluster 3 (Day 11) in mES cells
ECM PROTEIN
ACTIN BINDING
METABOLISM
CD2314
Bio
-
+
+
-
CD2314
Bio
-
+
+
-
CD2314
Bio
-
+
+
-
-
-
-
-
+
+
-
+
+
-
+
+
TRANSCRIPTION FACTORS
CD2314
Bio
-
+
+
-
CD2314
Bio
-
+
+
-
-
-
-
+
+
-
+
+
WNT SIGNALLING
CD2314
Bio
-
+
+
-
CD2314
Bio
-
+
+
-
-
-
-
+
+
-
+
+
