# Supplementary figures and images for: Comprehensive transcriptome analysis of mouse embryonic stem cell adipogenesis unravels new processes of adipocyte development
Source: Genome Biol. 2010 Aug 3;11(8):R80. doi: 10.1186/gb-2010-11-8-r80 (PMC2945782; doi:10.1186/gb-2010-11-8-r80)

Fig.S3, Billon *et al.*

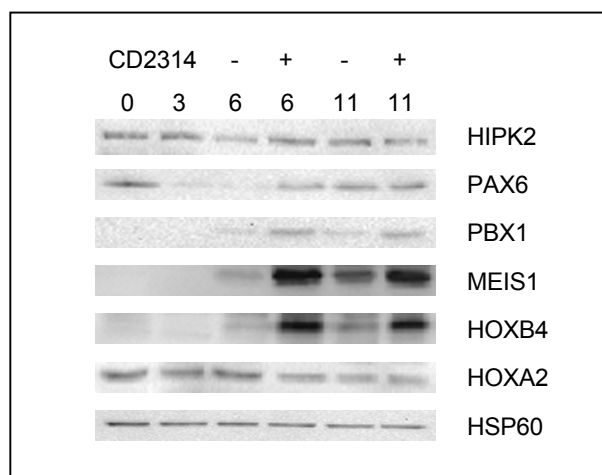

Supplement: Additional file 3 — Protein expression validation of MEIS1-associated transcriptional complex in mESCs. Cell extracts were generated at various time points before (day 0 and 3) or after (day 6 and 11) induction of adipocyte development by CD2314. The protein levels of HIPK2, PAX6, MEIS1 HOXB4, HOXA2, PBX1, and HSP60 were then assessed by conventional western blot analysis. Rabbit polyclonal antibodies were from Abcam (Paris, France; HIPK2, HOXB4, HOXA2) or Millipore (Molsheim, France; PAX6). Goat polyclonal antibody against HSP60 was from Santa Cruz Biotechnology, Inc. (Heidelberg, Germany) and was used to control for protein loading. Mouse antibodies against PBX1 and MEIS1 were generous gifts from M Cleary (Stanford University). [file gb-2010-11-8-r80-S3.PDF]
