# Supplementary material for: Comprehensive transcriptome analysis of mouse embryonic stem cell adipogenesis unravels new processes of adipocyte development
Source: Genome Biol. 2010 Aug 3;11(8):R80. doi: 10.1186/gb-2010-11-8-r80 (PMC2945782; doi:10.1186/gb-2010-11-8-r80)
Supplement: Additional file 4 — Primer pairs used for qPCR. [file gb-2010-11-8-r80-S4.PDF]

Figure S4, Billon et al.

gene	RNA Accession N°	forward primer (5'-3')	reverse primer (5'-3')
β-actin	NM_007393.1	ctgtgctatgttgccctggatt	gctcattgccgatggtgatc
calcrl	NM_018782.1	tgctctgtgaaggcgtttacct	ggcaggaagcagaggaaacc
cart1	NM_172553.2	gaggccagggtccaggttt	gtcgtacgtggcagcgaaat
cart1	NM_172553.2	gaggccagggtccaggttt	gtcgtacgtggcagcgaaat
cldn5	NM_013805.3	ccgctctcagagtccgttga	cgatgaaatctgagcgttcca
cobl	NM_172496.2	acgttgattgggtccttgaatg	cccgcacgacagccttct
col3a1	NM_009930.1	ctggaagccagaaccatgtca	gctgtgggcatattgcacaac
dact1	NM_021532.3	cagacggttgccccaaatc	gttgcccgtcagacaaagga
dcbld1	NM_025705.2	tcccggcatggttctttgt	ttgcaaagatccccattcca
dlk1	NM_010052.3	cgaaatagacgttcgggctt	tcgtactggcctttctccag
fabp4	NM_024406.1	cttcaaactgggcgtggaa	ctagggttatgatgctcttcacctt
gapdh	NM_008084.2	catggccttccgtgttccta	tgcctgcttcaccaccttct
hprt	NM_013556.2	gcctaagatgagcgcaagttga	aggcagatggccacaggacta
itga9	NM_133721.1	caccgtctacctcaaccaagga	ggaacccgtcatcatcaatgtc
lpl	NM_008509.2	gaggccagggtccaggttt	gccaggcaggaagcagaga
meox1	NM_010791.3	attatcccccaaccccgttt	gccaggcaggaagcagaga
nfIb	NM_008687.3	cttctccaccgagcagcaaa	cttgatctcgttcatgccatgtt
nr2f2	NM_009697.3	gcggaggaacctgagctacac	cccactttgaggcactttttga
pax6	NM_013627.3	ccagcttcaccatggcaaa	gcacgagtatgaggaggtctga
pbx1	NM_008783.2, NM_183355.2	ggcggaagagacggaatttc	tgccgcacttcttggctaac
polr2a	NM_009089.2	tcgaattgacttgcgtttcca	gcaaagttggctgacgattga
prxx2	NM_009116.1	ccgtggcaccaaacgaaa	gaggttgacacggcgagcta
pvlap	NM_032398.1	caaggaaacagtcatgcagcaa	gcggcgatgaagcgattata
rarβ	NM_011243.1	gcccaccatctccacttcct	gcaggcactgacgccatagt
sfrp1	NM_013834.2	tgaagaacggtgccgactgt	cccacttgtgaatggctgtca
sfrp2	NM_009144.1	ctggagtgcgaccgtttcc	cttggttttgcaggcttcaca
sfrp5	NM_018780.2	gaccaagatctgtgcccagtgt	aactttcggtccccgttgtc
shox2	NM_013665.1	ggcgaagtcggaccaatttt	attcctcgcgcatgaaagc
shox2	NM_013665.1	ggcgaagtcggaccaatttt	attcctcgcgcatgaaagc
sox18	NM_009236.2	gactcgaccggatgccacta	tgctctcttctggacaggacatg
spock3	NM_023689.2	cagtgcaccaaggcgttctt	tcccctgtcgcttctgaatg
stra8	NM_009292.1	gcctcaaagtggcaggtactga	gctgttgggattcccatcttg
tbox18	NM_023814.3	gtcccccatcaagcctgttc	ctcggaa acctttggcaaatg
tbp	NM_013684.2	gtgcacaggagccaagagtga	cacagctccccaccatgttc
tbx18	NM_023814.3	gtcccccatcaagcctgttc	ctcggaa acctttggcaaatg
tek	NM_013690.2	ccgtgctgttggcgtttc	cctgttaagggccagagttcct
wif1	NM_011915.1	aatggcccccttacacatga	cgcagcttgccaggtaaaatt
wnt2	NM_023653.4	cgggtcctcctccgaagtag	tggatcacaggagcaggacttt
